# Ribosome plasticity in glioblastoma: a multi-omics framework for future investigation

**DOI:** 10.3389/fgene.2025.1756452

**Published:** 2026-01-12

**Authors:** Dario Benelli, Christian Barbato, Carlo Cogoni

**Affiliations:** 1 Department of Molecular Medicine, Sapienza University of Rome, Rome, Italy; 2 Institute of Biochemistry and Cell Biology (IBBC), National Research Council (CNR), ‘Sapienza’ University, Rome, Policlinico Umberto I, Rome, Italy

**Keywords:** glioblastoma, multi-omics, ribosomal proteins, ribosome biogenesis, ribosome heterogeneity, translational regulation, tumour plasticity

## Abstract

Glioblastoma (GBM) remains a challenging tumour to mechanistically dissect, in part because of its capacity to adapt to hypoxia, metabolic imbalance and therapeutic pressure. Across cancer biology more broadly, attention has increasingly turned to ribosomal proteins (RPs). Although long regarded as stable structural components of the ribosome, several RPs show variation across tumour regions, stress states and differentiation trajectories. In some cancers, specific RPs have been mechanistically linked to selective translation or cell-state transitions, whereas in others the evidence remains largely associative. Overall, current observations leave open the question of whether RP variation reflects active regulatory roles or instead mirrors the broader physiological pressures experienced by malignant cells. In this mini review, we summarise what multi-omics approaches—including transcriptomics, proteomics and translatomics—currently reveal about RP regulation in GBM. Rather than making firm causal claims, we outline the main interpretations proposed so far, the uncertainties that complicate them and the conceptual gaps that keep the field open. Our aim is to provide a balanced and cautious overview that may help frame future work on how ribosomal components and the translational machinery could contribute to GBM plasticity.

## 1 Introduction

Glioblastoma (GBM) is often approached as an outlier among solid tumours—highly adaptive, metabolically stressed, and strikingly heterogeneous (Badr et al., 2020; Cortes Ballen et al., 2024; Singh et al., 2025). Yet one aspect that has begun to attract renewed interest is something far more fundamental: the ribosome. Historically regarded as a uniform and largely invariant molecular machine, the ribosome is now understood to exhibit multiple forms of heterogeneity (Catalanotto et al., 2023). Recent work has shown that ribosomal complexes can vary in their composition due to non-stoichiometric incorporation of ribosomal proteins, the presence of RP paralogs, differential rRNA modifications, and post-translational modifications of individual RPs—features that together may confer specialized translational functions or context-dependent selectivity (Gay et al., 2022). This conceptual shift has also reached the field of neuroscience, where several studies have shown that ribosomal proteins vary across neuronal subtypes, developmental stages, and even synaptic compartments (Shigeoka et al., 2019; Fusco et al., 2021; Dastidar and Nair, 2022; Gao and Wang, 2024). Techniques such as TRAP, RiboTag, ribosome profiling, single-nucleus RNA-seq and spatial transcriptomics have revealed that neurons regulate translation with a degree of precision and regional specificity that was previously underappreciated (Dougherty, 2017; Glock et al., 2021; Kilfeather et al., 2024). Within this framework, several observations illustrate how ribosomal variation operates in neural systems. For example, activity-dependent local translation in dendrites relies on ribosomes enriched for specific RPs (Shigeoka et al., 2019; Fusco et al., 2021). During development, additional regulated changes have been described for RPL10, RPS23 and other core components (Hetman and Slomnicki, 2019). Consistent with these findings, ribosomopathies affecting the same RPs produce well-defined neurological phenotypes (Brooks et al., 2014; Paolini et al., 2017). Collectively, these examples underscore that neural tissue is highly sensitive to alterations in ribosomal composition. These observations raise a fundamental question: how does ribosome heterogeneity mechanistically influence translation? Quantitative mass spectrometry studies in mouse embryonic stem cells have demonstrated that several core RPs are substoichiometric, with some present on only 60%–70% of actively translating polysomes, providing direct evidence that ribosomes of distinct compositions coexist and function within the same cell (Shi et al., 2017). Functionally, heterogeneous ribosomes appear to preferentially translate specific subsets of mRNAs through recognition of *cis*-regulatory elements in their untranslated regions (UTRs). Internal ribosome entry sites (IRESs) represent a key mechanism for such specialized translation: RPS25 is required for IRES-mediated initiation of both viral and cellular mRNAs, while RPL38 specifically enables IRES-dependent translation of a subset of *Hox* mRNAs critical for axial skeletal patterning (Landry et al., 2009; Kondrashov et al., 2011; Xue and Barna, 2012). In the case of *Hox* genes, a translation inhibitory element (TIE) near the 5′cap suppresses cap-dependent initiation, rendering these transcripts reliant on RPL38-containing ribosomes for their expression. Similarly, RPL24 facilitates translational re-initiation following upstream open reading frames (uORFs), thereby modulating the expression of developmentally regulated transcripts (Nishimura et al., 2005). Together, these findings establish that distinct ribosome compositions can coordinate the translation of functionally related gene networks, introducing an additional layer of post-transcriptional regulation with significant implications for cellular differentiation and tissue homeostasis (Xue and Barna, 2012; Genuth and Barna, 2018).

Beyond neural physiology, mounting evidence indicates that ribosomal protein dysregulation represents a general mechanism in cancer etiology across diverse tissue types (Aspesi and Ellis, 2019). Somatic mutations in RPL5 and RPL10 are recurrent in acute leukemias (De Keersmaecker et al., 2013), while RPS14 and RPS19 haploinsufficiency drives myelodysplastic syndromes (Ebert et al., 2008; Vlachos et al., 2008). Several ribosomal proteins (RPs) are over-expressed in solid tumours and in some cases this altered expression has been associated with more malignant cellular behaviour, such as increased proliferation or resistance to therapy. In gastric cancer, for example, RPS13 has been reported to promote tumour cell growth and both RPS13 and RPL23 have been implicated in chemoresistance, although their value as independent prognostic markers for patient outcome remains to be clarified (Shi et al., 2004). Similar associations have been described in other tumour types, including RPL36a in hepatocellular carcinoma (Kim et al., 2004), and RPL39 in circulating breast cancer cells (Dave et al., 2017). Importantly, these alterations are not mere passengers of malignant transformation but actively contribute to oncogenic processes through specialized translational programs that favor the expression of proliferation, survival, and stemness factors (Xue and Barna, 2012). This broader oncological context matters when discussing GBM, because it establishes ribosomal dysregulation as both a general principle of tumorigenesis and a mechanism with particular relevance in neural tissue, a microenvironment already primed for ribosome-mediated translational plasticity. Collectively, these examples, which span both healthy neural physiology and diverse malignancies, suggest that ribosomal protein dysregulation may represent a general oncogenic mechanism. This idea may be particularly relevant in GBM, where the documented sensitivity of neural tissue to ribosomal alterations appears to converge with the abnormal demands of aggressive tumor growth.

In this light, the patterns observed in GBM, such as regional differences in RP expression, phosphorylation, or incorporation into ribosomes (Shirakawa et al., 2020; Hide et al., 2022; Larionova et al., 2022), do not appear as isolated anomalies. Rather, they parallel mechanisms long recognized in neural development, plasticity, and disease. This does not prove that RP variation in GBM has functional consequences, but it strengthens the plausibility of such a hypothesis. At the same time, caution remains essential. Indeed, repeated patterns cannot automatically be taken as evidence of causality. Many changes in RP abundance could reflect metabolic or stress responses rather than dedicated regulatory mechanisms. Nonetheless, the parallels between neural physiology and GBM biology provide a relevant framework for exploring these questions.

## 2 Observations from multi-omics studies

Recent spatial transcriptomic profiling has revealed that the expression of several ribosomal proteins is non-uniform across the GBM microenvironment. Analyses of large-scale datasets, including IVY GAP, TCGA and multiple single-cell atlases, indicate that some ribosomal proteins show spatially patterned expression across functionally distinct regions of GBM, including hypoxic cores, infiltrative margins and perivascular niches (Puchalski et al., 2018; Neftel et al., 2019; Larionova et al., 2022). Among these, RPS27 displays a marked upregulation in GBM and represents one of the most consistently elevated ribosomal proteins across malignant compartments (Puchalski et al., 2018).

Compositional heterogeneity also emerges at the level of RNA processing, as alternative splicing of ribosomal protein paralogs—including RPL22 and RPL22L1—differs across tumour states and cellular phenotypes (Larionova et al., 2022), suggesting that ribosomal diversity in GBM may arise through multiple regulatory layers. Although these patterns are typically discussed within the tumour context, similar region-specific differences in RP abundance have long been recognised in the healthy brain, as noted earlier. The consistency between physiological neural heterogeneity and tumour-associated RP variation suggests that the ribosome may be inherently more dynamic in neural tissue than previously assumed.

Proteomics provides converging evidence for ribosomal plasticity in GBM. Quantitative datasets reveal pronounced mismatches between RP transcripts and their encoded proteins, as well as non-stoichiometric RP abundance across tumour regions (Stetson et al., 2020). Large-scale comparative proteomic studies further show that several RPs are consistently altered across independent GBM datasets, even when transcriptomic data fail to capture these shifts (Tribe et al., 2021). Together, these findings suggest that ribosomal composition in GBM is shaped by regulatory layers not fully visible at the mRNA level.

Beyond differences in RP abundance, post-translational modifications (PTMs) add a further layer of ribosomal variability in both neural tissue and cancer. In mammalian systems, multiple PTMs including phosphorylation, acetylation, methylation and ubiquitination modulate ribosome assembly, stability and selective translation. These regulatory mechanisms support the concept that ribosomes can deviate from strict stoichiometry or acquire specialised functions (Genuth and Barna, 2018). Experimental studies in cancer biology show that individual ribosomal proteins undergo regulated PTMs. For example, RPL26 is ubiquitinated and stabilised in ways that promote tumour progression (Gong et al., 2023). RPS3 undergoes phosphorylation that affects translational fidelity and mediates stress-responsive translation (Gao and Hardwidge, 2011; Poncová et al., 2019; Ahn et al., 2023). In addition, RPS27L participates in the DNA damage response through MDM2-dependent ubiquitination (Xiong et al., 2011). Although these modifications have not yet been comprehensively mapped in GBM, the existence of RP-PTMs in stressed or proliferative mammalian cells provides a mechanistic rationale for considering PTM-based ribosomal heterogeneity in the tumour context.

**FIGURE 1:**
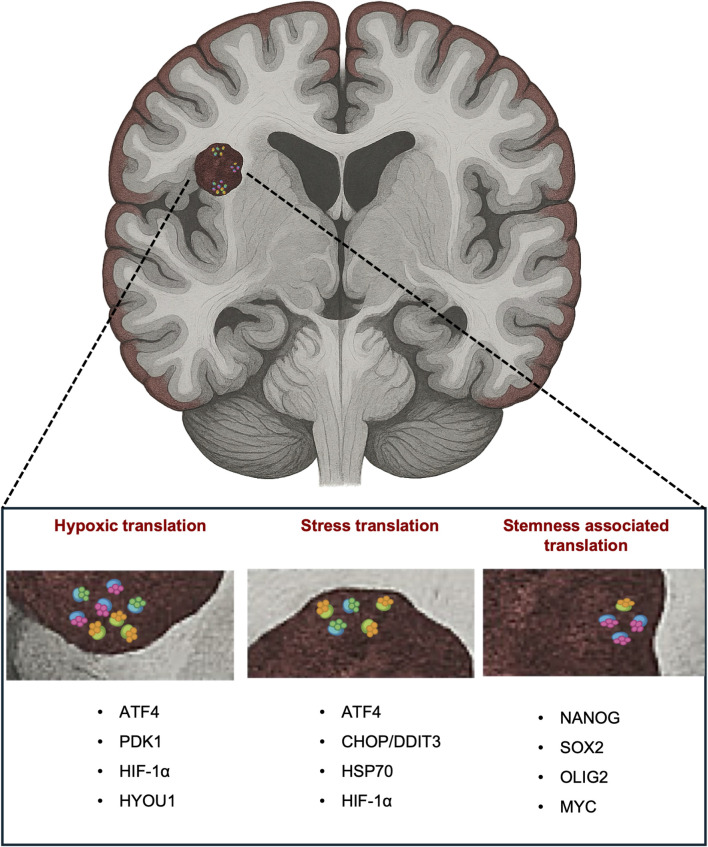
Spatially organized ribosomal heterogeneity in GBM. A coronal brain section illustrates a glioblastoma region enriched in ribosomes with diversified compositions. Enlarged insets highlight three representative microenvironments—hypoxic, stress-associated, and stemness-associated—each displaying distinct patterns and relative abundances of heterogeneous ribosomes. These spatial differences suggest that local microenvironmental pressures may contribute to region-specific translational programs within the tumor. At the bottom, the list of genes adjacent to each inset provides illustrative examples of mRNAs known to undergo selective translation under the corresponding growth conditions (e.g., ATF4, PDK1, HIF1A in hypoxia), highlighting how microenvironment-driven ribosomal states may influence the translational output of specific stress-responsive transcripts.

Ribosome profiling studies in GBM indicate a selective enhancement of stress-responsive mRNA translation, particularly under hypoxia or nutrient limitation. Many of these transcripts, including ISR-regulated mRNAs such as ATF4, are translated through non-canonical initiation mechanisms (e.g., uORF-mediated re-initiation), consistent with a stress-adapted translational program. (Gonzalez et al., 2014). Local translation in neurons provides an informative comparison, as dendritic ribosomes have been shown to translate specific mRNAs during synaptic plasticity and to exhibit RP compositions distinct from those in the soma (Puighermanal et al., 2017; Shigeoka et al., 2019; Glock et al., 2021). If neurons use ribosomal variation as part of their physiological regulatory repertoire, it becomes more plausible that GBM cells, which often adopt developmental or progenitor-like transcriptional states (Neftel et al., 2019), might deploy related mechanisms in pathological contexts. This does not prove functional specialisation within tumour ribosomes, but it strengthens the rationale for investigating it. Indeed, the patterns of RP dysregulation and selective translation observed across the GBM microenvironment could suggest a model where distinct RP-containing ribosomes act as translational filters to favor mRNAs associated with localized stress, hypoxia, or stemness-related conditions (Figure 1).

## 3 What perturbation experiments reveal—and what they can’t

Experimental perturbations of ribosomal proteins in GBM frequently produce strong phenotypes, although interpretation is complicated by the essential nature of these proteins. For example, silencing RPS15A in glioblastoma cells reduces proliferation and induces G0/G1 arrest, revealing a dependency on this RP in malignant glioma (Yao et al., 2016). Similar vulnerabilities have been documented in neural progenitors exposed to ribosomal stress. In human brain ribosomopathy models, hiPSC-derived neural progenitor cells carrying SNORD118 mutations exhibit impaired ribosome biogenesis, reduced global translation, decreased proliferation and increased apoptosis, resulting in defective cerebral organoid growth (Zhang et al., 2024). Studies in rodent neurons indicate that reducing the availability of specific ribosomal proteins triggers a ribosomal-stress–dependent activation of p53, which in turn perturbs neuronal growth and morphogenesis (Slomnicki et al., 2016; Hetman and Slomnicki, 2019). These findings suggest that developing neural cells are intrinsically sensitive to modest disturbances in ribosome function and that some vulnerabilities associated with RP imbalance may therefore be shared across malignant and non-malignant neural systems.

Overexpression experiments add additional complexity. Increasing RPS6 levels enhances sphere formation and stem-like features in glioblastoma models, with RPS6 expression particularly enriched in perivascular and perinecrotic niches where glioma stem-like cells predominate. Moreover, phosphorylated RPS6 levels are elevated in high-grade gliomas, suggesting potential context-dependent or extra-ribosomal roles (Shirakawa et al., 2020). Notably, in neurons several ribosomal proteins shuttle between cytoplasm and nucleus during synaptic maturation and can interact with chromatin or RNA-binding proteins outside of ribosomes, suggesting that ribosomal proteins can occupy multiple cellular compartments in neural contexts (Glock et al., 2021). Perturbations of ribosome biogenesis regulators also support this broader view. For example, inhibiting WDR12 reduces tumour growth *in vivo* and induces transcriptomic shifts consistent with partial differentiation (Li et al., 2020; Mi et al., 2021). Neural developmental studies suggest that even relatively modest disruptions in ribosome assembly may influence lineage specification or hinder aspects of neuronal maturation (Kondrashov et al., 2011; Slomnicki et al., 2016; Hetman and Slomnicki, 2019). Accordingly, when conceptually similar alterations are observed in GBM, it remains possible that the tumour may be re-engaging—or destabilising—regulatory programs that are normally active during neural development. Thus, when similar effects are observed in GBM, it remains possible that the tumour is re-engaging or destabilising regulatory programs that are active during normal neural development.

These parallels do not imply that GBM simply mirrors neural physiology, since tumours co-opt and distort underlying biological processes. Nonetheless, recognising that certain ribosomal behaviours have precedents in the healthy brain helps prevent premature dismissal of tumour-associated ribosomal protein variation as purely epiphenomenal.

## 4 Possible mechanisms and ongoing points of debate

Several hypotheses have been proposed to explain how RPs might influence GBM behaviour, and many resonate with mechanisms described in neural biology. For instance, the interaction between RPs and the MDM2–p53 axis is well established in other cellular systems (Kałużyńska, 2025; Lindström, 2025). Neural progenitors with ribosomal imbalance often activate similar stress pathways, suggesting a conserved link between ribosome integrity and genome surveillance (Slomnicki et al., 2018; Hetman and Slomnicki, 2019). Whether this axis retains functional significance in GBM depends heavily on the tumour’s p53 status, but the underlying logic is biologically plausible.

Selective translation is another area where tumour and neural literature intersect. Under hypoxia or nutrient deprivation, cap-dependent initiation becomes inefficient, increasing reliance on IRES-mediated translation (Holcik and Sonenberg, 2005). Neurons also rely on non-canonical initiation during synaptic plasticity and stress adaptation, including internal ribosome entry site (IRES)-mediated initiation (Pinkstaff et al., 2001), upstream open reading frame (uORF)-dependent translational control (Costa-Mattioli et al., 2009; Hacisuleyman et al., 2024), and near-AUG or non-AUG initiation events (Lee et al., 2024) and some RPs appear to modulate this process (Shigeoka et al., 2019; Fusco et al., 2021; Dastidar and Nair, 2022). In GBM, hypoxic translation has been reported to involve alternative cap-dependent initiation mechanisms, including the use of eIF4E2 and eIF4G3 in place of the canonical eIF4E/eIF4G complex (Uniacke et al., 2012; Kelly et al., 2018), while IRES-mediated translation has also been described for specific stress-responsive transcripts (Holcik and Sonenberg, 2005; Gonzalez et al., 2014). By contrast, a role for ribosomal protein variation in modulating IRES-dependent translation in GBM remains speculative and is currently supported mainly by mechanistic evidence from neuronal systems rather than by direct demonstrations in glioblastoma (Kapur et al., 2017; Shigeoka et al., 2019; Fusco et al., 2021).

Additional data connect RPs to DNA repair, chromatin organisation or RNA stability (Hegde et al., 2004; Coléno-Costes et al., 2012; Gentilella et al., 2017; Yang et al., 2019). In neurodevelopmental disorders linked to RPL10 (and possibly RPS23), defects in ribosome function often coincide with genome-maintenance defects or dysregulated transcription, raising the possibility that similar mechanisms may involve other ribosomal proteins (Thevenon et al., 2015; Paolini et al., 2017). Reports describing nuclear localisation of RPL22L1 in glioblastoma, together with studies showing phosphorylated RPS6 in specific tumour microenvironments, suggest that some ribosomal proteins may perform functions outside the ribosome under certain conditions, although these observations should be interpreted cautiously in light of the available evidence (Shirakawa et al., 2020; Lindström, 2025).

## 5 Convergence, divergence and open questions

Despite substantial methodological differences between transcriptomic, proteomic and translatomic studies, several points of convergence emerge. RP variation in GBM frequently aligns with microenvironmental gradients such as hypoxia, metabolic imbalance or proximity to the vasculature. Comparable gradients influence RP expression in the healthy brain, including during cortical development and within specialised neuronal compartments. Similarly, stem-like or therapy-resistant GBM populations often display RP patterns distinct from more differentiated tumour cells, mirroring how neural progenitors and mature neurons maintain different translational profiles. At the same time, important divergences and uncertainties remain. It is not yet clear whether tumour-associated RP changes represent adaptive regulatory mechanisms, secondary consequences of stress, or a mixture of both. The extent to which alternative isoforms or post-translational modifications create ribosomes with distinct biochemical or translational properties also remains unresolved, despite suggestive parallels from neuronal systems. Technical variation adds an additional layer of complexity, as differences in sample composition, sequencing depth or analytical pipelines can influence RP measurements.

Viewed against the backdrop of neural physiology—where ribosomal diversity is increasingly recognised as functionally relevant—these open questions underline both the promise and current limitations of interpreting RP heterogeneity in GBM. Clarifying which aspects reflect meaningful regulatory processes, and which instead reflect contextual or technical noise, remains a central challenge for the field (Box 1).

BOX 1 **Outstanding Questions.**

- Do changes in RP composition actively shape translational selectivity in GBM, as they do in neural systems, or do they mainly reflect broader cellular stress?
- Can RP isoforms or post-translational modifications generate ribosomes with distinct functional properties in tumours, similar to those observed during neural development or synaptic plasticity?
- How does spatial RP heterogeneity relate to ecological niches within GBM, and do these patterns mirror physiological gradients in neural tissue?
- To what extent might RP-associated DNA repair functions influence therapy response, given their known relevance in neurodevelopmental disorders?
- Are there context-specific dependencies tied to RP plasticity that could provide therapeutic leverage in GBM?

## 6 Discussion and future directions

Across the observations summarised in this mini-review, a recurring theme is that ribosomal variation in GBM appears to arise through multiple regulatory layers, yet the functional significance of these patterns remains incompletely defined. Transcriptomic and proteomic datasets reveal non-uniform RP abundance across tumour regions, while perturbation experiments show that altering specific RPs or ribosome-biogenesis factors can shift proliferation, differentiation markers or stress responses. At the same time, examples from neural development and ribosomopathies demonstrate that similar sensitivities to ribosome imbalance exist in non-malignant neural systems, suggesting that at least some features observed in GBM may reflect broader principles of ribosomal regulation in the brain rather than tumour-specific mechanisms. These parallels, however, also highlight the central uncertainty: whether RP differences in GBM actively contribute to lineage plasticity and adaptation, or whether they mainly mirror the physiological pressures the tumour experiences. Current datasets often allow both interpretations, and the complexity of ribosome function makes it difficult to exclude confounding effects such as altered proliferation, metabolic stress or changes in cell-state composition. Moreover, many datasets capture abundance rather than ribosome incorporation, leaving open the question of how frequently RP variation results into changes in translational output.

Looking ahead, several approaches may help address these gaps. A priority will be establishing whether regionally enriched ribosome variants are functionally active; that is, whether mRNAs showing increased translation in specific GBM areas, as identified by ribosome profiling, are preferentially loaded onto these variant ribosomes. Demonstrating such selective mRNA-ribosome pairing would be fundamental to establishing the biological significance of ribosomal heterogeneity in distinct tumour contexts. Second, integrating ribosome profiling with spatially resolved proteomics could clarify whether region-specific RP patterns correlate with selective translation *in situ*. Third, methods distinguishing ribosome-bound from unbound RP pools may assist in determining which variations reflect *bona fide* ribosomal heterogeneity. Fourth, perturbations targeted to defined neural-like or stem-like GBM subpopulations could help separate direct ribosomal effects from broader changes in tumour composition. Finally, comparisons with developmental models of neural ribosome regulation may provide a useful reference for distinguishing conserved physiological programs from tumour-specific rewiring.

Together, these directions underscore that while current evidence does not definitively establish specialised ribosome function in GBM, it does justify further investigation. A clearer picture of how translational machinery is regulated across GBM states may ultimately refine our understanding of tumour plasticity and identify new points of vulnerability.

### 6.1 Therapeutic perspectives targeting ribosomes and translation in GBM

Given the growing interest in ribosomal plasticity and translational regulation in glioblastoma, an important open question concerns whether these processes can be therapeutically exploited. At present, no approved therapies selectively target individual ribosomal proteins (RPs) in GBM, and there is no direct evidence that RP heterogeneity itself represents a druggable vulnerability. Instead, most experimental efforts have focused on broader perturbations of ribosome biogenesis or translation control, rather than on specific ribosomal compositions. In preclinical glioma models, inhibition of RNA polymerase I–dependent rRNA synthesis has been reported to impair tumour cell proliferation and induce nucleolar stress, suggesting that GBM cells may exhibit a heightened sensitivity to disruptions in ribosome production (Zisi et al., 2023). Similarly, pharmacological interference with ribosome biogenesis regulators, such as WDR12, reduces growth and stem-like properties in glioma models, although these effects are not specific to ribosomal protein diversity *per se* and likely reflect a general dependency on ribosome output (Li et al., 2020).

Beyond ribosome biogenesis, several studies have proposed that GBM cells rely on context-dependent modes of translation initiation, particularly under hypoxic or stress conditions (Holcik and Sonenberg, 2005; Gonzalez et al., 2014; Montiel-Dávalos et al., 2023). Targeting components of the translational machinery—including initiation or elongation factors—has therefore been explored as a complementary strategy, with some compounds showing activity in GBM cell systems (Porcù et al., 2023). However, these approaches generally affect global or stress-adapted translation programs, rather than selectively modulating ribosomes with distinct RP compositions. Importantly, issues such as blood–brain barrier penetration, therapeutic index and tumour heterogeneity remain major obstacles for translating these findings into clinical applications. Taken together, current evidence suggests that while the ribosome and the translational apparatus represent areas of therapeutic interest in GBM, the specific concept of targeting ribosomal protein heterogeneity remains largely unexplored experimentally. In this context, the emerging parallels between ribosomal regulation in neural physiology and tumour biology may be most valuable not as an immediate therapeutic roadmap, but as a guide for identifying conditions under which translational dependencies become exposed. Establishing whether distinct ribosomal states correspond to selective translational outputs in GBM will be a critical prerequisite for evaluating whether ribosome-associated vulnerabilities can be exploited in a more targeted manner.
